# The Shear Bond Strength of Resin-Based Luting Cement to Zirconia Ceramics after Different Surface Treatments

**DOI:** 10.3390/ma16155433

**Published:** 2023-08-02

**Authors:** Grzegorz Sokolowski, Agata Szczesio-Wlodarczyk, Małgorzata Iwona Szynkowska-Jóźwik, Wioleta Stopa, Jerzy Sokolowski, Karolina Kopacz, Kinga Bociong

**Affiliations:** 1 Department of Prosthodontics, Medical University of Lodz, 251 Pomorska St., 92-213 Lodz, Poland; 2 University Laboratory of Materials Research, Medical University of Lodz, Pomorska 251, 92-213 Lodz, Poland; 3 Faculty of Chemistry, Institute of General and Ecological Chemistry, Lodz University of Technology, Zeromskiego 116, 90-543 Lodz, Poland; 4 Department of General Dentistry, Medical University of Lodz, Pomorska 251, 92-213 Lodz, Poland; 5 “DynamoLab” Academic Laboratory of Movement and Human Physical Performance, Medical University of Lodz, ul. Pomorska 251, 92-216 Lodz, Poland; 6 Warsaw Medical Academy, Ludwika Rydygiera 8, 01-793 Warszawa, Poland

**Keywords:** zirconia, surface, treatment, Zircos-E, etching, primers, resin, cements, SBS, bond, adhesion

## Abstract

Due to its unique properties, zirconia is increasingly being used in dentistry, but surface preparation for bonding is difficult because of its polycrystalline structure. This study aimed to determine the effect of a new etching technique (Zircos-E) on Ceramill Zi (Amann Girrbach). The effect of etching and the use of primers (Monobond Plus and MKZ Primer) on the bond strength of zirconia with resin cement (NX3) was assessed. Shear bond strength was evaluated after storage in water for 24 h and after thermal aging (5000 thermocycling at 5 °C/55 °C). A scanning electron microscope (Hitachi S-4700) was used to evaluate the surface structure before and after the Zircos-E system. The roughness parameters were assessed using an SJ-410 profilometer. The etched zirconia surface is more homogeneous over the entire surface, but some localized forms of erosion exist. The etching of zirconia ceramics caused changes in the surface structure of zirconia and a significant increase in the shear bond strength between zirconia and resin cement. The use of primers positively affects the adhesion between resin cement and zirconia. Aging with thermocycler significantly reduced the shear bond strength, with one exception—sandblasted samples with MKZ Primer. Standard ceramic surface preparation, involving only alumina sandblasting, does not provide a satisfactory bond. The use of etching with the Zircos-E system and primers had a positive effect on the strength of the zirconium–resin cement connection.

## 1. Introduction

The most popular ceramics used in dental practice are lithium disilicate, silica, alumina, leucite, and zirconia-based materials. However, zirconia is favored both in research and in the clinic. The number of research publications related to this type of ceramic nearly tripled in the years 2012–2019 compared to 2007–2011 [1]. This may be related to its remarkable mechanical strength and aesthetic properties. Some oxides, such as CaO, MgO, Y_2_O_3_, and CeO_2_, are added to stabilize the high-temperature zirconia phase (tetragonal) at normal temperatures. Yttrium-stabilized zirconia (Y-TZP) is most often used in dentistry due to its combination of high strength and optimum optical properties [2]. Sintered Y-TZP has been shown to have superior flexural strength (900–1200 MPa), fracture resistance (˃2000 N), and fracture toughness (9–10 MPa·m^0.5^) in comparison with other conventional ceramics, including alumina-reinforced and lithium-disilicate-based ceramics [3,4]. Because of its high biocompatibility, corrosion resistance, and light weight, zirconia is a material that is frequently used in biomedical applications [5]. In addition, prosthetic restorations can be made using zirconia with the assistance of computer-aided design (CAD) and computer-aided manufacturing (CAM) systems in order to obtain a final product that is perfectly integrated into the physiognomy of each patient [6].

Success in prosthetic treatment is not only related to selecting the appropriate material for the reconstruction, but requires the creation of appropriate adhesion. Unfortunately, due to the crystalline structure of zirconia and the lack of a silicon dioxide (silica) phase, conventional methods of preparing dental ceramics (including etching with acidic solutions and applying silane coupling agents) do not result in sufficient bond strength in restorations [3]. Different treatments of the zirconia surface have been studied in recent years. One of the basic methods used in prosthetics is sandblasting. In this method, the sandblaster emits alumina particles (which are most often used) under a certain pressure. The energy of the ejected particles erodes the ceramic surface, increasing its roughness and wettability [7]. Of note is the fact that the air abrasion of zirconia can also lead to surface deformation, i.e., plastic deformation and/or melting of the surface, micro-cracks, gaps, driving of the abrasive grain into the surface, etc. [8]. In order to avoid damage to the zirconia surface, the sandblasting protocol with a small particle size (30 µm) at moderate pressure (2.5 bar) is recommended [9,10]. Some reports show that high-pressure (0.4 MPa) air abrasion may have a negative impact on the biaxial flexural strength of Y-TZP [11]. In addition, the presence of a monoclinic phase was reported in zirconia materials after sandblasting and etching. The content was up to 5% [12,13,14]. However, a meta-analysis carried out by Aurélio I. L at al. [15] indicated that the flexural strength of Y-TZP is improved by airborne-particle abrasion, regardless of the particle size, the parameters of blasting (pressure and time), and the presence of aging. 

Nevertheless, the surface of zirconia should be prepared before bonding with any cement in order to promote micromechanical interlocking mechanisms. The durability and stability of the bonds between ceramics and adhesives can be improved because the physical bonds are not susceptible to the degradation caused by environmental factors, such as humidity [8]. Some authors have studied the possibility of applying zirconia surface primers (e.g., 10-methacryloyloxydecyl dihydrogen phosphate (MDP)) or silanes to promote better adhesion. Studies have shown that some primers achieved a high and durable bond strength. It is most likely that only primers containing monomers with phosphate ester groups can bond directly to metal oxides [16,17]. Considering the internal structure of yttrium-stabilized zirconia, its surface cannot be activated with traditional etching methods, for example, 9.5% hydrofluoric acid for 60 s. Various acid treatments have been proposed for zirconia. Most of the proposed acid treatments assume the use of a high concentration of acid (>30%) and prolonged action (with a minimum of several minutes) and, very often, the treatments are conducted at an elevated temperature [12,18,19,20]. The Zircos-E system (M & C Dental, Seoul, Republic of Korea) is a commercially available product that is dedicated to zirconia etching. It contains hydrofluoric acid (HF), hydrochloric acid (HCl), sulfuric acid (H_2_SO_4_), nitric acid (HNO_3_), and phosphoric acid (H_3_PO_4_). This patented technology uses ionization to create a microporous surface, which may improve bonding strength [21]. In the literature, only limited studies use this method for zirconia surface conditioning compared with abrasion and primer treatment [22]. 

The first null hypothesis was that etching with the Zircos-E system or using primers (MKZ Primer or Monobond Plus) does not affect bond strength. The second was that the surface topography of Ceramill Zi (Amann Girrbach AG, Koblach, Austria) is not affected by etching with the Zircos-E system (M & C Dental, Seoul, Republic of Korea).

## 2. Materials and Methods

The monolithic zirconia CAD-CAM blocks (Ceramil Zi, Amann Girrbach AG, Koblach, Austria) were sectioned using a milling device (Ceramill Motion 1, Amann Girrbach AG, Koblach, Austria). Subsequently, the specimens were sintered according to the manufacturer’s instructions. The samples were cylinder-shaped with a diameter of 10 mm and a height of 10 mm. The surface was ground under water coolant with 180 grit, followed by 320 and 600 grit silicon carbide paper, to unify sample surfaces. Prepared specimens were sandblasted with 50 μm Al_2_O_3_ particles under a pressure of 2 bar. After that, the specimens were washed under running water for 30 s and then air-dried. Half of the specimens were etched for 30 min at 30 °C using Zircos-E Etching Solution (M & C Dental, Seoul, Republic of Korea). Zircos-E Etching Solution contains hydrofluoric acid (HF), hydrochloric acid (HCl), sulfuric acid (H_2_SO_4_), nitric acid (HNO_3_) and phosphoric acid (H_3_PO_4_). After the etching process, the samples were cleaned with running water for 3 min and then with steam.

According to the conducted surface preparation, the specimens were divided into 6 groups (*n* = 22), as follows:

- Control: No chemical treatment was conducted.
- Primer MKZ: MKZ Primer (Bredent, Senden, Germany) was applied according to the manufacturer’s instructions.
- Primer Monobond Plus: Monobond Plus (Ivoclar Vivadent, Schaan, Lichtenstein) was applied according to the manufacturer’s instructions.
- Etched: No additional treatment after etching was conducted.
- Etched + Primer MKZ: After etching, MKZ Primer was applied according to the manufacturer’s instructions.
- Etched + Primer Monobond Plus: After etching, Monobond Plus was applied according to the manufacturer’s instructions.

OptiBond Solo Plus (bonding agent) was applied according to the manufacturer’s instructions on prepared samples. Next, composite cement (NX3, Kerr, Brea, CA, USA) was packed into a plastic mold with a diameter of 3 mm and a height of 3 mm and light-cured for 20 s using a light-curing unit (1200 mW/cm^2^, the CURE—TC-01, Spring Health Products, Norristown, PA, USA).

**Figure A1:**
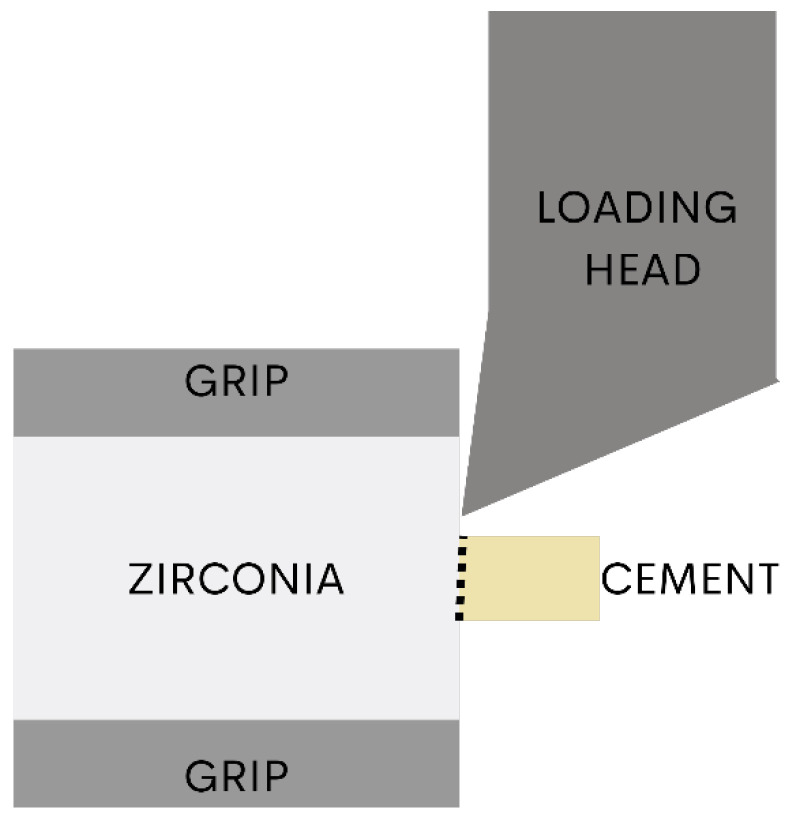
Illustration of the shear bond strength test.

Eleven samples of each group were stored at 37 °C in distilled water. After 24 h storage, the samples were shear-loaded (Figure A1, Appendix A) to fracture at 2 mm/min crosshead speed using a universal testing machine of 5 kN maximum load cell capacity (Zwick-Roell Z005, Zwick-Roell, Ulm, Germany), with a distance between the crosshead and substrate of smaller than 1 mm.

Another 11 samples of each group were subjected to thermocycling between 5 °C and 55 °C for 5000 cycles, with a 20 s dwell time (THE 1200, SD Mechatronic, Feldkirchen-Westerham, Germany). After that, the shear bond strength was evaluated.

The roughness of the control and etched samples was investigated using a compact surface roughness tester SJ-410 (Mitutoyo, Kawasaki, Japan). Surface roughness for each study group was measured five times in different directions, from which the mean values were calculated. The evaluation length was 4 mm. Six roughness profile parameters were compared: roughness average (Ra), the average maximum height of the profile (Rz), maximum profile peak height (Rp), maximum profile valley depth (Rv), mean spacing of profile irregularities (Sm), retention volume (Vo).

Control and etched materials’ surface topography was investigated with a scanning electron microscope energy-dispersive spectroscopy (SEM–EDS) microscope Hitachi S-4700 (Tokyo, Japan). The images were made at a magnification of 1000×, 5000× and 15,000×.

Shear bond strength data were analyzed by the Shapiro–Wilk test to assess the normality of the distributions. Based on the results, the Kruskal–Wallis test with multiple comparisons of mean ranks was applied. Data obtained from roughness measurements were subjected to Student’s *t*-test to compare parameters (Ra, Rz, Rp, Rv, Sm, Vo) of control and etched samples. All analyses were performed using Statistica version 13 software (StatSoft, Kraków, Poland). The value α = 0.05 was adopted as significant. 

## 3. Results

**Figure 1:**
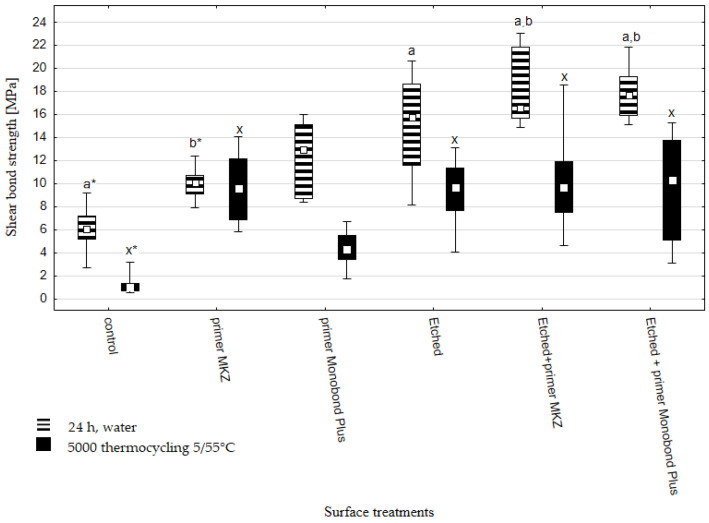
Box and whisker plot of shear bond strength (median values with minimum and maximum value) of resin-based luting cement to zirconia ceramics after different surface treatments. Shear bond strength was evaluated after storage in water for 24 h and after thermal aging (5000 thermocycling at 5 °C and 55 °C). a Significant difference at the level of *p* ≤ 0.05 were found between results assigned with the letter with asterisk and the same letter.

The results of shear bond strength after 24 h in water and thermocycling are presented in Figure 1. All failures were determined to be adhesive based on visual observations.

The highest median value of shear bond strength evaluated after 24 h in water was 17.7 MPa (etched+ Primer Monobond Plus), and the lowest was 6.1 MPa (control) (Figure 1). The use of thermal aging (5000 thermocycles at 5 °C and 55 °C) resulted in a significant decrease in the bond strength for almost all tested groups. Based on the Kruskal–Wallis’s test, a statistically significant difference was found with *p*-value ≤ 0.0000. 

According to multiple comparisons of mean ranks for 24 h, water series, statistically significant differences were found between:

- Control vs. Etched (*p* = 0.000434);
- Control vs. Etched + primer MKZ (*p* = 0.000001);
- Control vs. Etched + primer Monobond Plus (*p* = 0.000001);
- Primer MKZ vs. (b) Etched + primer MKZ (*p* = 0.003873);
- Primer MKZ vs. Etched + primer Monobond Plus *(p* = 0.004601).

According to multiple comparisons of mean ranks for 5000 thermocycling, 5/55 °C series, statistically significant differences were found between:

- Control vs. (x) primer MKZ (*p* = 0.000114);
- Control vs. Etched (*p* = 0.000069);
- Control vs. Etched + primer MKZ (*p* = 0.000059);
- Control vs. Etched + primer Monobond Plus (*p* = 0.00075).

**Figure 2:**
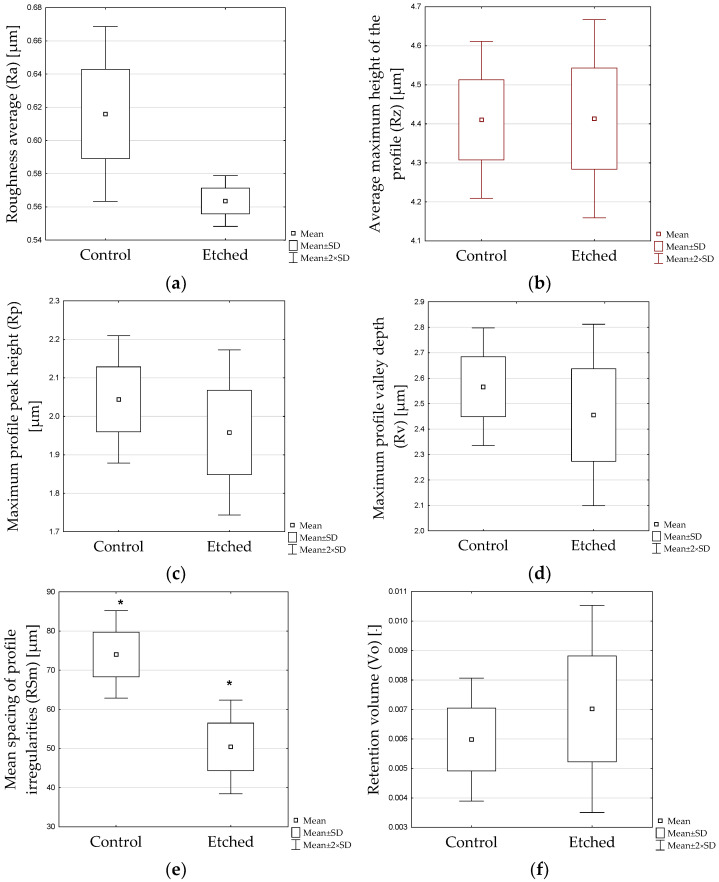
Roughness parameters of control and etched Ceramill Zi. (**a**) roughness average (Ra), (**b**) the average maximum height of the profile (Rz), (**c**) maximum profile peak height (Rp), (**d**) maximum profile valley depth (Rv), (**e**) mean spacing of profile irregularities (RSm), (**f**) retention volume (Vo). The results assigned with the asterisk show significant difference at the level of *p* ≤ 0.05.

Results of roughness measurements are presented in Figure 2a–d.

According to Student’s *t*-test, statistically significant differences were found in the mean spacing of the profile irregularities (RSm); parameter (*p* < 0.05).

**Figure 3:**
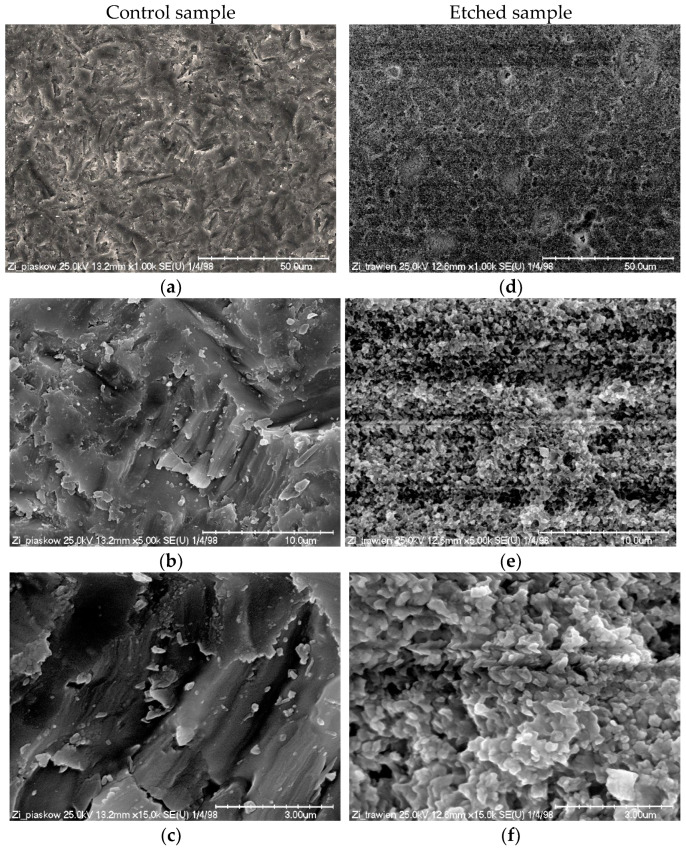
Scanning electron microscopy (SEM) micrographs of control ((**a**–**c**)—sandblasted, 50 μm Al_2_O_3_, 2 bar) and etched ((**d**–**f**)—sandblasted, 50 μm Al_2_O_3_, 2 bar and etched 30 min. at 30 °C using Zircos-E Etching Solution) Ceramill Zi at 1000× ((**a**,**d**), scale bar—50 µm), 5000× ((**b**,**e**), scale bar—10 µm) and 15,000× ((**c**,**f**), scale bar—3 µm) magnification. The figures demonstrate increased brightness.

**Figure 4:**
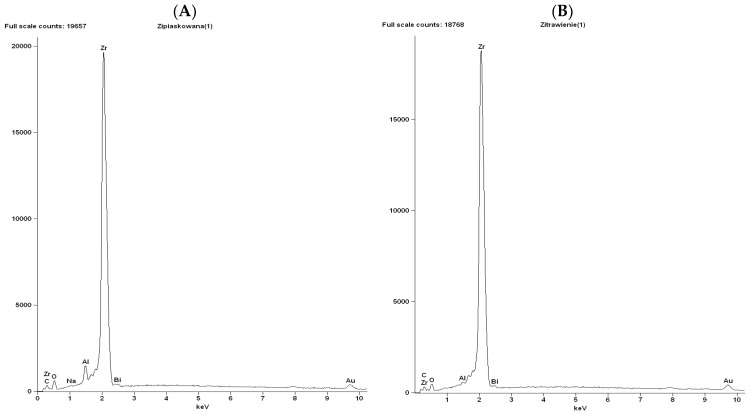
Chemical composition analysis of Ceramill Zi (**A**) control (sandblasted, 50 μm Al_2_O_3_, 2 bar), (**B**) etched (sandblasted, 50 μm Al_2_O_3_, 2 bar and etched 30 min. at 30 °C using Zircos-E Etching Solution). Data were obtained by energy-dispersive spectroscopy (EDS).

The surface topography of control and etched samples at a magnification of 1000×, 5000×, and 15,000× is presented in Figure 3a–f. A chemical composition analysis of the tested materials is given in Figure 4A,B.

The images obtained with the SEM evaluation show clear differences between the surface topography of the two materials. Both samples show an isotropic structure; however, the etched sample is more homogeneous, with regularly and evenly distributed crystal grains. Ceramill Zi is composed of zirconium oxide and the signals corresponding to zirconium (Zr) and oxygen (O) are mainly visible in the analyzed spectra (Figure 4A,B). The spectrum of the control sample shows a clear signal from aluminum (Al); the amount of this element in the sample subjected to etching is decreased.

## 4. Discussion

Manufacturing a zirconia prosthetic restoration poses a challenge, as it requires the creation of an appropriate connection with resin cement. Traditional etching methods are ineffective because the material is polycrystalline and has no silica phase compared to conventional ceramic materials. Our findings, based on analyses of shear bond strength, suggest that the etching (Zircos-E system) and presence of primers (MKZ Primer or Monobond Plus) influence the adhesion of resin cement (NX3). SEM micrographs showed differences in the structure after etching. Therefore, the null hypothesis can be rejected. 

All samples were first sandblasted (control sample). This treatment caused isotropic roughening and irregularities formed on the surface (Figure 3). The structure after the etching process is also irregular and resembles erosion. However, the structure after etching is more homogeneous over the entire surface. There seem to be some localized forms of erosion on the etched sample—pitting. Comparable structure observations were made by A. Sales et al. [21], who also used the Zircos-E system for their research. Similar differences between sandblasted samples and those additionally etched with a strong acid solutions were also observed in other studies [12,23,24]. Proper surface treatments may promote the micromechanical interlocking of resin cement by creating fitting roughness and increasing surface energy [11,25,26,27]. Profilometers are commonly used to characterize roughness. They enable the determination of certain parameters of the evaluated surface. The roughness average parameter, Ra, due to its relative repeatability and stability, is most frequently used. However, more roughness parameters should be determined to characterize material structure because Ra does not fully characterize the surface of the material [28,29]. In the present study, six parameters were analysed (Figure 2). Some changes in roughness parameters were observed. Ra for the sandblasted surface (control sample) was 0.6160 ± 0.0601 µm, whereas the value for the sandblasted and etched surface was 0.5636 ± 0.0174 µm; however, this difference was statistically insignificant. The peaks and valleys characteristics of both samples were comparable (Figure 2b–d). A significant difference was observed for the mean spacing of profile irregularities between the control (74.04 ± 12.74 µm) and etched (50.42 ± 13.62 µm) sample (Figure 2e). Smaller distances between irregularities on the surface can cause zirconia micro-retentions, resulting in increased bond strength. There are only a few studies evaluating the surface roughness after sandblasting and etching. Some reports show that the value of Ra is lower than or close to that of sandblasted samples in materials subjected to etching [30]. In contrast, in some reports, Ra values after etching are higher [20]. Compared to the untreated (polished) sample, etching causes surface roughening [19,24,31,32,33]. It should be noted that not every surface development is positively reflected in bond strength [34]. In addition, in the case of bond strength, not only is the height of the roughness important, but other parameters should also be considered, e.g., roughness, width, frequency and regularity. Our study confirms that etching creates a more homogenous surface structure and enlarges surface area. This surface condition more easily contributed to a higher bond strength [35]. In the present study, the shear bond strength (SBS) of zirconia with resin cement was evaluated after different surface treatments: air abrasion, air abrasion and etching, and a combination of those with two primers (MKZ Primer, Monobond Plus). SBS results showed statistical differences between control and etched samples (with or without primers) (Figure 1).

Treatment with the Zircos-E system improved the bond strength compared to the sandblasted samples. Observed changes in surface topography and smaller distances between irregularities on the surface result in the better micromechanical interlocking of resin cement. A similar dependency can be found in other studies [21]. In the mentioned study, the connection (assessed with micro-shear bond strength) of resin cement with zirconia improved when Zircos–E was used, juxtaposing this with sandblasting or a surface without any preparation (28.48 MPa vs. 22.66 MPa or 18.96 MPa for opaque zirconia and translucent 28.12 MPa vs. 25.36 MPa or 22.82 MPa, respectively) [21]. Besides, the best results were observed when the zirconia surface was both sandblasted and etched. In a study conducted by Cho et al. [22], the shear bond strength between zirconia after air-abrasion, acid etching (nitric acid–hydrofluoric acid) and tribochemical silica-coating and resin cement were measured. The bond strength in the etching group was higher compared to other studied groups but the authors did not determine how durable this connection was. In a study carried out by Sadid-Zadeh R. et al. [36], all prepared specimens (air-abraded with 50 µm Al_2_O_3_; etched with Zircos-E; air-abraded and then etched with Zircos-E; etched with Zircos-E and then sandblasting) were subjected to thermocycling for 1000 cycles between 5 °C and 55 °C. Similar results (around 10 MPa) were obtained regardless of the surface preparation method. The authors suggested that an additional study should be performed, nothing the potentially hazardous nature of such an acid solution [36]. In another study, the SBS (after 10,000 thermocycles) of etched zirconia surface bonded with enamel by resin cement was insignificantly lower than the connection with sandblasted zirconia, and both surface preparation methods appeared to be more effective than those observed in the control (non-prepared ultra-translucent zirconia) [37]. The authors claimed that lower bond strength observed for Zircos-E is attributed to high viscosity of resin cement which prevents flow in the irregularity of the etched surface [37].

In our study, the application of selected primers also increased bond strength. This increase was, however, statistically insignificant. MKZ primer contains 3-Methacryloyloxypropyl-trimethoxysilan and 10-Methacryloyloxydecyl dihydrogen phosphate. Monobond Plus has three different functional methacrylates—silane, phosphoric, and sulfide methacrylate. Silanes can react with hydroxyl groups of silica and the methacrylate of resin cement, creating quite a strong connection with inorganic ceramics. However, in zirconia, there is no silica phase, so this bonding cannot occur with silane substances of used primers [38,39]. However, the used primers contain some acidic monomers with a phosphate ester group that can bond directly to zirconium oxide and enhance the wettability of the ceramic surface [40,41]. In addition, sandblasting and etching not only develop a surface that is conducive to the formation of micro-retentions, but also contribute to the formation of hydroxyl groups, which may chemically react with phosphate monomers [25]. Some studies have shown that primers enhance the bond strength of resin cement to zirconia ceramics [41,42]. The important aspect are sandblasting and the type of cement used (conventional vs. self-adhesive) [43]. 

Long-term water storage and thermocycling are common methods of assessing bond durability [44,45,46]. In thermocycling aging, degradation is caused not only by the reaction of water molecules with bond interface/resin cement, but also by the contraction and expansion of the materials due to temperature variations. It is worth noting that zirconia and resin cement have different thermal coefficients, which may result in greater stress accumulation at the interface. In addition, the larger the bonding interface in clinical conditions in comparison with laboratory tests, the greater the observed degradation effect. In the present study, after 5000 thermocycles, which correspond to six months of service life as 10,000 cycles are supposed to correspond to 12 months of service [47], shear bond strengths decreased significantly in comparison with initial values (24 h, water 37 °C). This result corresponded to the results obtained in other studies showing that bonding between zirconia ceramics and resin luting agents is not as stable after thermocycling [34]. The minimum value of bond resin cement, which guarantees the safe exploitation of ceramic under clinical conditions, is about 10–13 MPa [48,49]. A similar value of the minimum bond strength of the resin-to-restoration interface (not lower than 10 MPa) was proposed by Behr M. et al. [50] for resin-bonded fixed partial dentures from the Maryland type. Except for zirconia, which was only air-abraded (control) before and after thermocycling, and when Monobond was used after thermocycling, the value of all connections with cement in our study are close to this value.

In summary, our study confirms that zirconia can be etched. It also shows an increase in shear bond strength. The observed changes in SEM images may be related to the corrosion of zirconia grains. More chemically reactive atoms around the crystal boundaries dissolve faster than those inside the crystal, which leads to the formation of irregular grooves around the crystals and grain size reductions [51]. It was shown that HF dissolves zirconium oxide and yttrium oxide but, during this process, fluoride, oxide, and hydroxide complexes are formed [52]. The resulting structure is more homogeneous compared to the sandblasted samples. Additionally, there are some porosities that can contribute to the formation of better microretentions following micromechanical interlocking with adhesive. Furthermore, it has been shown that acid contributes to the formation of -OH groups on the zirconia surface. Hydroxyl groups can be used for chemical reaction with primer components and chemically modify the zirconia surface, as well as enhancing the adhesion of resin cement [53,54]. It has also been found that there is a direct chemical connection between the phosphate ester group of adhesive monomers and zirconia oxide [55]. Not only are ionic interactions between 10-MDP and zirconia observed, hydrogen bonding can be observed too [56].

Further studies on different types of cement and variations in the etching protocol (time, temperature) would provide a more detailed insight into the effect of the Zircos-E Etching solution on zirconia to improve bond strength. A comparison of the use of silane alone would allow for an evaluation of the effectiveness of this substance in comparison with primers dedicated to zirconium (containing other monomers). Testing mechanical properties, such as hardness and flexural strength after such aggressive etching, should be evaluated in the future for zirconia ceramic. In addition, an evaluation of the effect of certain treatments on zirconia phases with the use of X-ray diffraction and FITR would be an interesting contribution to research on zirconia.

## 5. Conclusions

Considering the limitations of the present study, the following conclusions can be drawn:

- Use of the Zircos-E system influences the shear bond strength between zirconia and resin cement. In consequence, SBS is significantly higher than that observed in the control group (only sandblasted zirconia) and when only a primer (after sandblasting) is used.
- Etching with the Zircos-E system leads to changes in the surface structure—a lower average roughness and mean spacing of profile irregularities. Etching also reduces the amount of alumina element (from air abrasion) on the zirconia surface.
- Use of primers preceded with etching with the Zircos-E system positively affects the adhesion between resin cement and zirconia.
- Aging with thermocycler significantly reduced the shear bond strength, except for sandblasted samples with MKZ Primer, which stayed on the same level ~10 MPa, before and after aging.

## Data Availability

Data are available in a publicly accessible repository Zenodo at https://zenodo.org/record/8215149 (last accessed 4 July 2023).
